# Preserved ratio impaired spirometry with or without restrictive spirometric abnormality

**DOI:** 10.1038/s41598-023-29922-0

**Published:** 2023-02-20

**Authors:** Shinichiro Miura, Hiroshi Iwamoto, Keitaro Omori, Kakuhiro Yamaguchi, Shinjiro Sakamoto, Yasushi Horimasu, Takeshi Masuda, Shintaro Miyamoto, Taku Nakashima, Kazunori Fujitaka, Hironobu Hamada, Akihito Yokoyama, Noboru Hattori

**Affiliations:** 1 Department of Molecular and Internal Medicine, Institute of Biomedical & Health Sciences, Hiroshima University, 1-2-3 Kasumi, Minami-Ku, Hiroshima, 734-8551 Japan; 2 Department of Infectious Diseases, Hiroshima University, Hiroshima, Japan; 3 Department of Respiratory Medicine and Allergology, Kochi University, Kochi, Japan

**Keywords:** Chronic obstructive pulmonary disease, Epidemiology

## Abstract

Preserved ratio impaired spirometry (PRISm) is defined by reduced FEV_1_ with a preserved FEV_1_/FVC ratio; some individuals with PRISm can also have restrictive ventilatory abnormality. The aim of this study was to clarify clinical features of restrictive and non-restrictive PRISm. In total, 11,246 participants (mean, 49.1 years; range, 35–65 years) from five healthcare centres were included in this study. We evaluated baseline characteristics of participants with restrictive PRISm (FEV_1_/FVC ≥ 0.7, FEV_1_ < 80% and FVC < 80%) and non-restrictive PRISm (FEV_1_/FVC ≥ 0.7, FEV_1_ < 80% and FVC ≥ 80%), and airflow obstruction (FEV_1_/FVC < 0.7). We examined the longitudinal risk of developing airflow obstruction by comparing spirometry results at baseline and 5 years post-baseline among 2141 participants. Multivariate analysis demonstrated that a history of asthma or smoking could constitute an independent risk factor for non-restrictive PRISm, and that non-restrictive PRISm was independently associated with the risk of developing airflow obstruction. In contrast, female sex, advanced age, and high BMI, but not history of asthma or smoking, were risk factors for restrictive PRISm. Restrictive PRISm was not associated with the development of airflow obstruction. In conclusion, our results indicate that PRISm can be categorized according to the presence or absence of restrictive abnormality. Non-restrictive PRISm, which does not meet the conventional criteria of obstructive and restrictive ventilatory abnormalities, may be a precursor of chronic obstructive pulmonary disease and merits increased monitoring.

## Introduction

Preserved ratio impaired spirometry (PRISm) is defined by reduced FEV_1_ (< 80% of predicted value) with a preserved FEV_1_/FVC ratio (≥ 0.7), and its prevalence is between 3 and 20%^1–4^. PRISm is reported to be a risk factor in the development of COPD and increased respiratory symptoms and mortality; it is also associated with the risk of cardiovascular disease^1–6^. Additionally, previous reports show that PRISm is associated with various characteristics including sex, smoking, age, metabolic syndrome, systemic inflammation, exposure to dust, history of tuberculosis, and asthma^1,2,7–9^. These observations suggest that PRISm might include heterogenous groups of people with increased risk of respiratory disease and systemic comorbidities.

Restrictive ventilatory abnormality, together with obstructive abnormality, is a widely used spirometric criteria^10^. Restrictive abnormality is associated with increased respiratory symptoms^11–13^ and increased risk of various comorbidities, such as cardiovascular disease, metabolic syndrome, hypertension, stroke, and diabetes^14–19^. PRISm can be divided into two groups according to the presence or absence of a restrictive abnormality (FEV_1_/FVC ≥ 0.7 and FVC < 80% predicted)^15^. There may be differences in clinical features between PRISm with or without restrictive abnormality, but there has been no study to investigate them separately.

The aim of this study was to clarify the differences in clinical features between restrictive and non-restrictive PRISm. In this study, we divided individuals with PRISm into two subgroups according to the presence or absence of a restrictive abnormality and examined potential risk factors and longitudinal risk of developing airflow obstruction.

## Materials and methods

### Study design and study population

**Figure 1:**
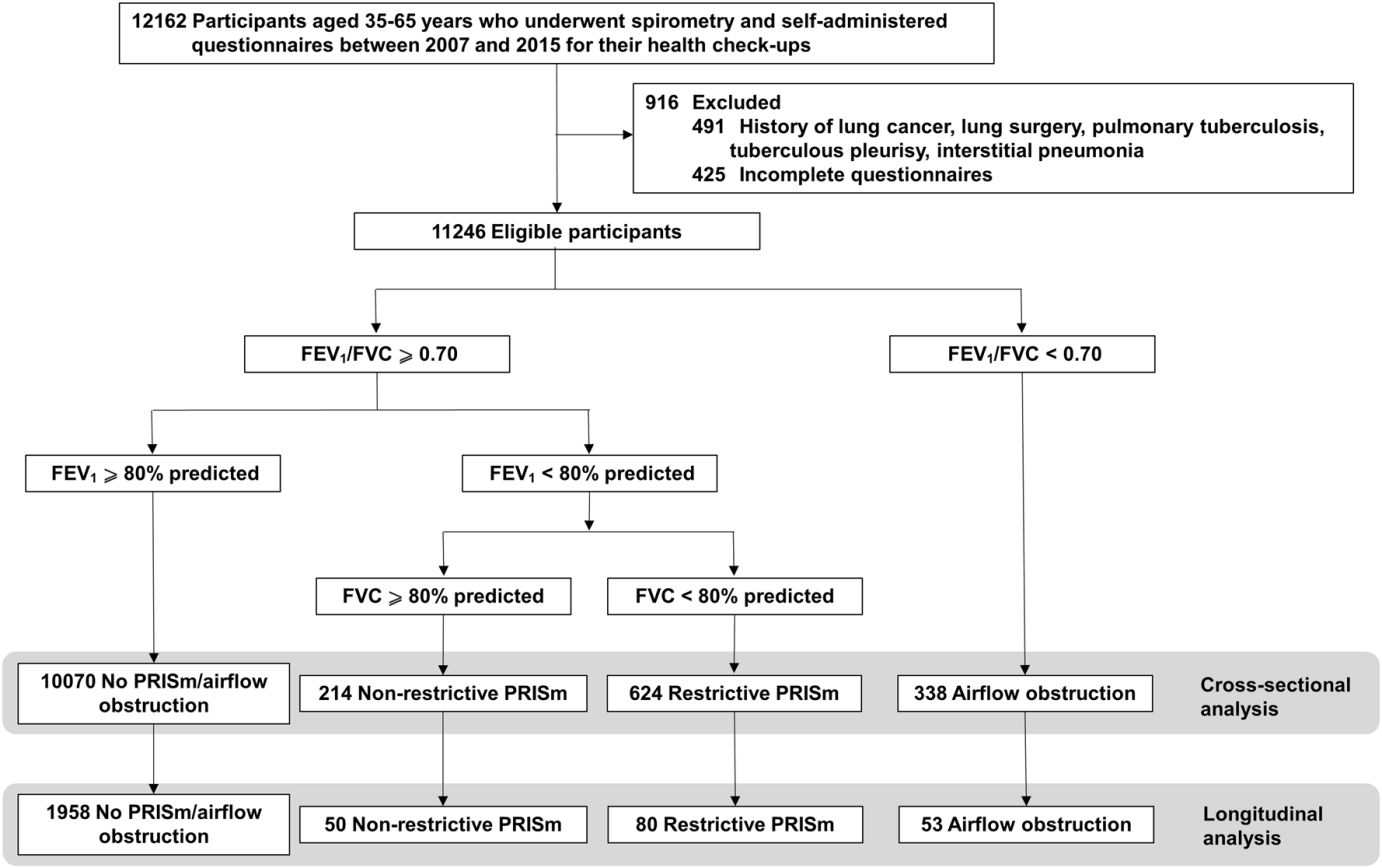
Flow diagram of the participant selection process in this study. FEV_1_—forced expiratory volume in 1 s; FVC—forced vital capacity; PRISm—preserved ratio impaired spirometry.

This was a survey study of participants who visited one of five healthcare centers in Hiroshima, Japan, between 2007 and 2015 for their annual health check-ups, including spirometry. In total, 12,162 participants aged 35–65 years were enrolled. Participants with histories of lung cancer, lung surgery, pulmonary tuberculosis, tuberculous pleurisy, interstitial pneumonia and participants who submitted incomplete questionnaires were excluded from the analysis (n = 916). The remaining 11,246 study participants were eligible for the cross-sectional analysis (Fig. 1). Of the 11,246 participants, 2141 participants were followed-up with five years post-baseline; these were included in the longitudinal analysis. All participants were informed of the aims of this study and that their participation was entirely voluntary and anonymized. This study was conducted in accordance with the ethical standards established in the Helsinki Declaration of 1975. The Medical Ethics Committee of Hiroshima University approved this study and waived the requirement for obtaining the participants’ signed informed consent (E-M699-1).

### Spirometry and classifications

Pre-bronchodilator pulmonary function was measured using portable spirometers (Chest-AC33, Chest HI-801; Chest Co., Tokyo, Japan; FUDAC-77, SP-350; Fukuda Denshi Co., Tokyo, Japan). The Japanese reference values for pulmonary function were used^20^.

**Figure 2:**
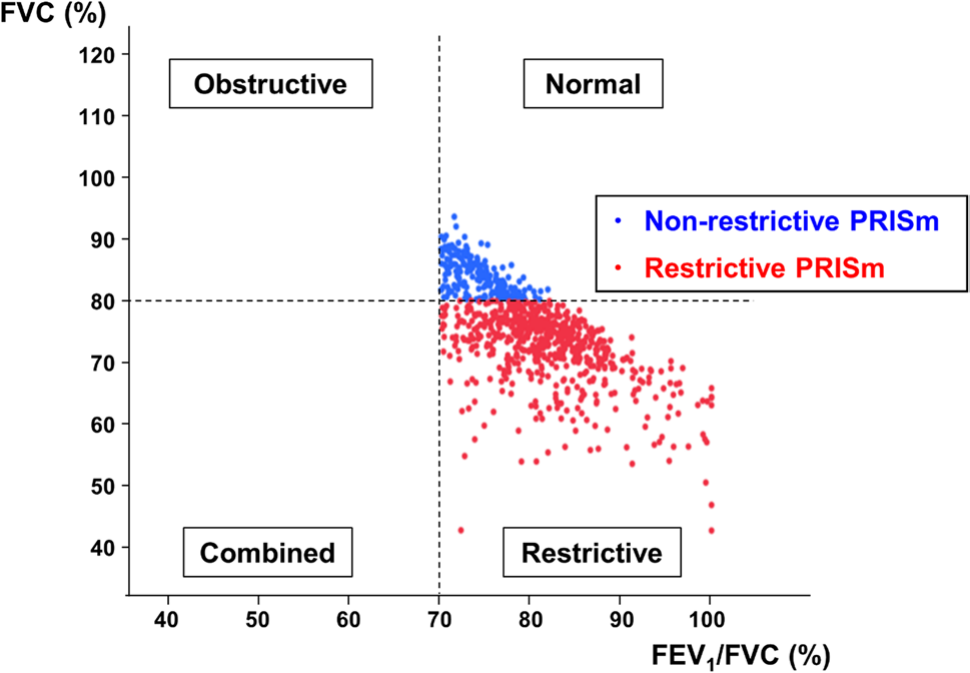
Distribution of spirometric results of participants with restrictive and non-restrictive PRISm. FEV_1_—forced expiratory volume in 1 s; FVC—forced vital capacity; PRISm—preserved ratio impaired spirometry.

The participants were categorized as follows based on their baseline lung function measurements: no PRISm/airflow obstruction (FEV_1_/FVC ≥ 0.7 and FEV_1_ ≥ 80%; n = 10,070), airflow obstruction (FEV_1_/FVC < 0.7 independent of FVC values; n = 338) and PRISm (FEV_1_/FVC ≥ 0.7 and FEV_1_ < 80%; n = 838). Individuals with PRISm were classified according to the presence or absence of restrictive abnormality; these groups were non-restrictive PRISm (FEV_1_/FVC ≥ 0.7, FEV_1_ < 80% and FVC ≥ 80%; n = 214) and restrictive PRISm (FEV_1_/FVC ≥ 0.7, FEV_1_ < 80% and FVC < 80%; n = 624) (Fig. 2).

### Questionnaire

The details of the self-administered questionnaires have been described previously^21,22^. Smoking habits, underlying respiratory or cardiac disease, exposure to dust or asbestos, and respiratory symptoms were investigated.

### Statistical analyses

Comparisons among groups were performed by using the chi-square test for categorical variables and the Kruskal–Wallis test followed by the Bonferroni correction for continuous variables. Univariate and multivariate logistic regression analyses were performed to investigate the clinical predictors of non-restrictive PRISm, restrictive PRISm and airflow obstruction in the cross-sectional analysis. Sex, age, body mass index (BMI), smoking status, and history of asthma were used as independent variables in the multivariate analyses. In the longitudinal analysis, univariate and multivariate logistic regression analyses were performed to examine whether non-restrictive PRISm or restrictive PRISm was a risk factor for development of airflow obstruction. Risk ratio was adjusted for age, sex, BMI, and pack-year. All data analyses were performed using JMP statistical software version 15.1.0 (SAS Institute Inc., Cary, NC, USA).

## Results

### Baseline characteristics and clinical predictors of the participants in the restrictive and non-restrictive PRISm

The participants with restrictive PRISm were more likely to be older, female, and obese when compared with the participants without PRISm/airflow obstruction (Table 1). The proportion of ever smokers with ≥10 pack-years was significantly higher in the non-restrictive PRISm group than in the restrictive PRISm and no PRISm/airflow obstruction groups. The proportion with a history of asthma was significantly higher in the non-restrictive PRISm than in the no PRISm/airflow obstruction group, but not in the restrictive PRISm group. In the participants with restrictive PRISm, the incidence of cough was significantly higher when compared with the participants without PRISm/airflow obstruction, and there was also a trend toward a higher incidence of breathlessness (*p* < 0.10). In the participants with non-restrictive PRISm, there was a trend toward a higher incidence of cough, phlegm and breathlessness when compared with the participants without PRISm/airflow obstruction. The mean values of %FEV_1_ and %FVC were significantly higher, but FEV_1_/FVC were significantly lower, in the non-restrictive PRISm group than in the restrictive PRISm group (Table 1).

**Table 1:** Baseline characteristics of the participants in the cross-sectional analysis.

	No PRISm/airflow obstruction (n = 10,070)	Non-restrictive PRISm (n = 214)	Restrictive PRISm (n = 624)	Airflow obstruction (n = 338)
Characteristics				
Male, n (%)	7762 (77.1)	167 (78.0)	442 (70.8)*	293 (86.7)*
Age (years)	49.0 ± 6.9	49.2 ± 6.7	50.1 ± 7.0*	52.0 ± 7.1*
BMI (kg/m^2^)	23.3 ± 3.2	23.7 ± 3.5	23.9 ± 4.3*	22.9 ± 2.8
BMI ≥ 25, n (%)	2675 (26.6)	61 (28.5)	214 (34.3)*	62 (18.3)*
BMI ≥ 30, n (%)	329 (3.3)	8 (3.7)	48 (7.7)*	4 (1.2)
Smoking status, n (%)				
Never smoker	4652 (46.2)	76 (35.5)	308 (49.4)	102 (30.2)
Ever smoker with < 10 pack-years	1118 (11.1)	20 (9.3)	40 (6.4)	31 (9.2)
Ever smoker with ≥ 10 pack-years	4300 (42.7)	118 (55.2)*¶	276 (44.2)*	205 (60.6)*
Exposure to dust, n (%)	708 (7.0)	10 (4.7)	28 (4.5)	20 (5.9)
Cardiac disease, n (%)	148 (1.5)	5 (2.3)	11 (1.8)	5 (1.5)
Respiratory symptoms, n (%)				
Cough	1020 (10.1)	29 (13.6)	93 (14.9)*	75 (22.2)*
Phlegm	1208 (12.0)	36 (16.8)	73 (11.7)	75 (22.2)*
Breathlessness	2561 (25.4)	64 (29.9)	185 (29.6)	126 (37.3)*
History of asthma, n (%)	679 (6.7)	30 (14.0)*	56 (9.0)	95 (28.1)*
Lung function measurements				
FEV_1_ (L)	3.14 ± 0.59	2.43 ± 0.36*¶	2.23 ± 0.39*	2.52 ± 0.65*
%FEV_1_	99.6 ± 11.0	77.1 ± 2.3*¶	73.1 ± 5.5*	78.2 ± 15.5*
FVC (L)	3.83 ± 0.75	3.27 ± 0.49*¶	2.74 ± 0.50*	3.86 ± 0.88
%FVC	97.8 ± 11.2	83.8 ± 2.9*¶	72.2 ± 5.9*	96.0 ± 15.5
FEV_1_/FVC (%)	82.4 ± 5.4	74.4 ± 2.8*¶	81.9 ± 6.0*	65.0 ± 6.1*

*BMI* body mass index; *FEV*_*1*_ forced expiratory volume in 1 s; *%FEV*_*1*_ percent predicted FEV_1_; *FVC* forced vital capacity; *%FVC* percent predicted FVC; *PRISm* preserved ratio impaired spirometry.

Variables are presented as mean ± SD or No. (%).

*P < 0.0125 for comparison with No PRISm/airflow obstruction, ¶*P* < 0.0125 for comparison with Restrictive PRISm.

Mann–Whitney *U*-test for continuous and chi-square for categorical variables.

Multivariate logistic analysis showed that history of asthma and smoking were independently associated with non-restrictive PRISm, whereas being of the female sex, having a more advanced age and having a higher BMI were independent risk factors for restrictive PRISm (Table 2).

**Table 2:** Univariate and multivariate logistic analyses investigating the risk factors of non-restrictive PRISm, restrictive PRISm and airflow obstruction.

Variable	Non-restrictive PRISm			Restrictive PRISm			Airflow obstruction		
	OR	95% CI	*p* value	OR	95% CI	*p* value	OR	95% CI	*p* value
Univariate analysis									
Male (vs. female)	1.06	0.76–1.47	0.726	0.71	0.59–0.85	< 0.001*	1.97	1.44–2.71	< 0.001*
Age (per 10 years)	1.01	0.83–1.22	0.958	1.24	1.10–1.40	< 0.001*	1.85	1.58–2.18	< 0.001*
BMI	1.03	0.99–1.07	0.146	1.05	1.03–1.07	< 0.001*	0.96	0.93–0.99	0.015*
Ever smoking with ≥ 10 pack-years (vs. never or ever smoking with < 10 pack-years)	1.60	1.21–2.10	< 0.001*	1.03	0.87–1.21	0.750	2.04	1.63–2.55	< 0.001*
Asthma (vs. healthy control)	2.00	1.35–2.97	< 0.001*	1.20	0.91–1.60	0.200	5.18	4.04–6.64	< 0.001*
Multivariate analysis									
Male (vs. female)	0.73	0.50–1.07	0.105	0.55	0.44–0.67	< 0.001*	1.51	1.06–2.17	0.024*
Age (per 10 years)	0.97	0.79–1.18	0.738	1.26	1.12–1.42	< 0.001*	1.91	1.61–2.26	< 0.001*
BMI	1.03	0.99–1.07	0.176	1.07	1.04–1.09	< 0.001*	0.92	0.89–0.96	< 0.001*
Ever smoking with ≥ 10 pack-years (vs. never or ever smoking with < 10 pack-years)	1.78	1.30–2.45	< 0.001*	1.17	0.97–1.41	0.109	1.72	1.34–2.21	< 0.001*
Asthma (vs. healthy control)	2.04	1.37–3.03	< 0.001*	1.16	0.87–1.57	0.311	6.21	4.79–8.06	< 0.001*

*BMI* body mass index; *CI* confidence interval; *OR* odds ratio; *PRISm* preserved ratio impaired spirometry.

*P < 0.05 logistic regression analysis.

### Transitions of lung function categories and risk factors for the development of airflow obstruction (longitudinal analysis)

**Figure 3:**
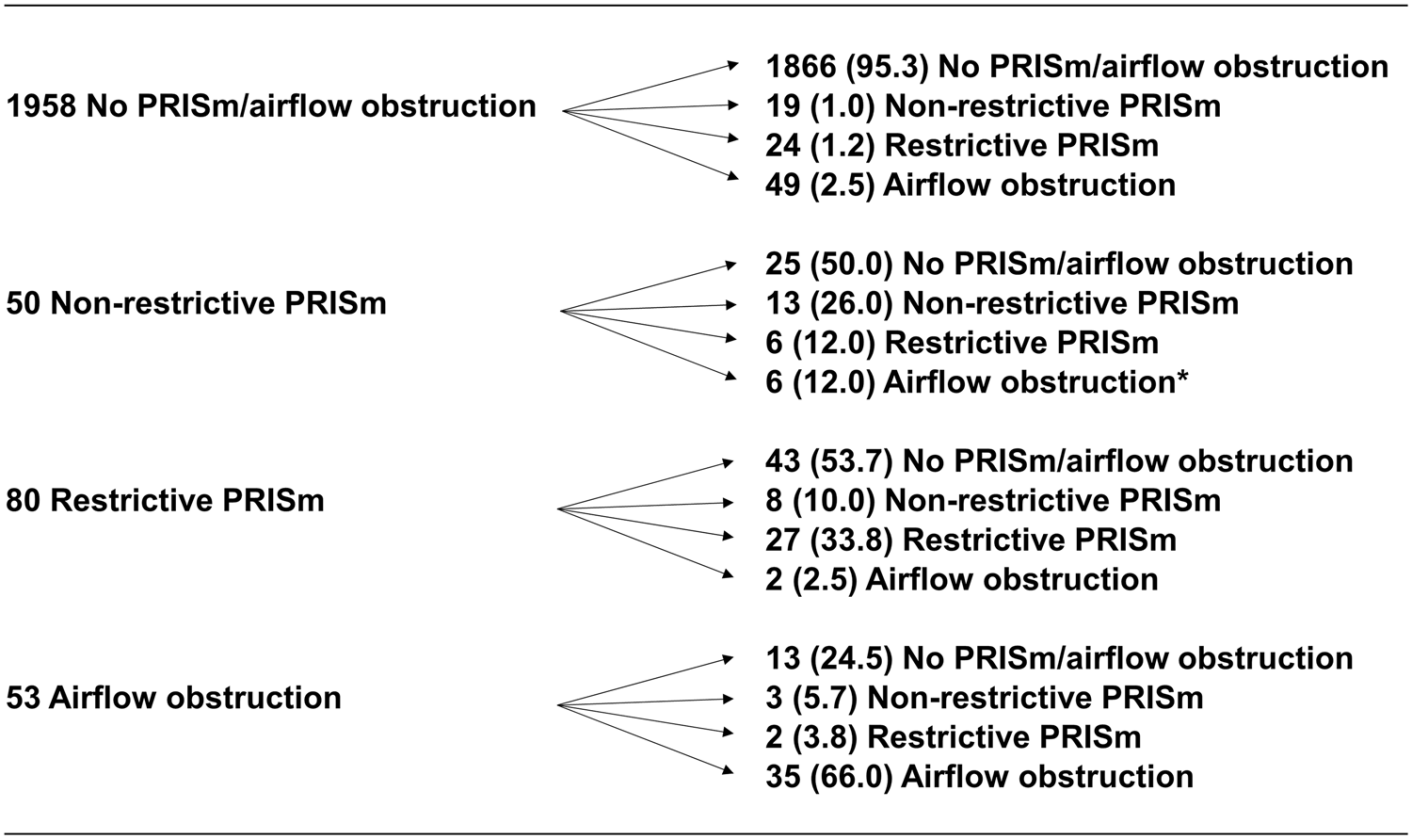
Transitions of lung function categories among participants between first visits and visits after five years. Variables are presented as No. (%). *P < 0.017 versus no PRISm/airflow obstruction by chi-square test. PRISm—preserved ratio impaired spirometry.

The characteristics of the participants in the longitudinal analysis were similar to those of participants in the cross-sectional analysis (Supplementary Table S1). Figure 3 shows the transitions of lung function categories among participants between their first visits, and their visits five years later. A higher proportion of the participants with non-restrictive PRISm transitioned to airflow obstruction compared with the participants without PRISm/airflow obstruction (12.0% and 2.5%, *p* < 0.017). No significant differences were observed in the proportion of transition to airflow obstruction between the restrictive PRISm and no PRISm/airflow obstruction groups. In participants with restrictive and non-restrictive PRISm, about half transitioned to the no PRISm/airflow obstruction category. Supplementary Figure S1 visually shows the transitions of lung function categories among participants with restrictive and non-restrictive PRISm using the conventional criteria of obstructive and restrictive ventilatory abnormalities. A quarter of participants with non-restrictive PRISm and half of those with restrictive PRISm transitioned to other categories. Univariate and multivariate logistic analysis showed that non-restrictive PRISm, but not restrictive PRISm, was independently associated with the development of airflow obstruction (Table 3; adjusted risk ratio, 4.47; 95% CI, 1.66–12.01; *p* = 0.003).

**Table 3:** Risk ratio, adjusted risk ratio, and 95% confidence interval (CI) for development of airflow obstruction in 5 years.

Baseline spirometry category	Risk ratio	95% CI	*p* value	Adjusted risk ratio*	95% CI	*p* value
No PRISm/airflow obstruction	1			1		
Non-restrictive PRISm	5.31	2.16–13.05	< 0.001	4.47	1.66–12.01	0.003
Restrictive PRISm	0.99	0.24–4.18	0.999	0.94	0.22–3.98	0.930

*BMI* body mass index; *PRISm* preserved ratio impaired spirometry.

*Risk ratio was adjusted for age, sex, BMI, and pack-year.

## Discussion

In the present study, we investigated the clinical features and longitudinal trajectory of lung function for individuals with restrictive and non-restrictive PRISm. Multivariate analysis of background demographics demonstrated that history of asthma and smoking were independently associated with non-restrictive PRISm, while female sex, advanced age, and high BMI were associated with restrictive PRISm. Longitudinal analysis of lung function showed that non-restrictive PRISm, but not restrictive PRISm, was independently associated with development of airflow obstruction. These results indicate that PRISm can be divided into two subgroups according to the presence or absence of restrictive spirometric abnormalities, and non-restrictive PRISm may be associated with increased risk of developing COPD.

The present study demonstrated that non-restrictive PRISm was independently associated with the development of airflow obstruction. Several longitudinal studies have shown that PRISm is associated with the development of airflow obstruction^2–4,6,23^. In the COPDGene study, approximately 25% of the current or ex-smokers with a spirometric finding of PRISm developed airflow obstruction five years later^2^. In a population-based cohort study at Rotterdam, 33% of individuals with PRISm developed airflow obstruction^4^. Marott et al. reported that individuals with PRISm between 20 and 40 years old experienced an increased risk of hospital admission for COPD in a 15-years long study^6^. The present study showed that non-restrictive PRISm, but not restrictive PRISm, is associated with an increased risk of developing airflow obstruction in 5 years. While restrictive PRISm can be classified into restrictive spirometric pattern, non-restrictive PRISm may be classified into normal spirometric pattern by the conventional criteria of obstructive and restrictive ventilatory abnormalities. Non-restrictive PRISm, which is more likely to be underestimated than restrictive PRISm in clinical practice, may be a precursor of COPD and may merit increased monitoring.

The present study also showed that there is a difference in the risk factors for restrictive PRISm and those for non-restrictive PRISm. A history of asthma and smoking were independent risk factors for non-restrictive PRISm, and female sex, advanced age, and high BMI were independent risk factors for restrictive PRISm. Previous population studies have shown that history of asthma and smoking are associated with obstructive ventilatory abnormality, and obesity is associated with restrictive abnormality^14,15,21,24–28^. Two large population-based European cohorts: ECRHS and SAPALDIA, reported that the incidence of history of asthma and heavy smoker was lower and the incidence of obesity was higher in individuals with restrictive ventilatory abnormality than in those with obstructive abnormality^15^. The TESAOD study showed that individuals with restrictive ventilatory abnormality were more likely to be female, to be non-smokers, and to not have had asthma, and to have lower IgE levels than those with obstructive abnormality^14^. The results of the present study indicate that non-restrictive PRISm had similar features to obstructive abnormality, while restrictive PRISm had similar features to restrictive abnormality.

Previous studies have reported that there are several subtypes of PRISm^1,4^. Wan et al. reported that PRISm was divisible into three subgroups through cluster analysis in observational cross-sectional study: “COPD-subtype,” “Restrictive-subtype,” and “Metabolic-subtype”^1^. The Rotterdam study reported that PRISm encompassed at least three distinct clinical groups: one with progression to COPD, a second with high cardiovascular burden and early death, and a third with persistent PRISm and normal age-related lung function decline^4^. These previous reports indicated that individuals with PRISm may be clinically heterogeneous, but the criteria for classifying subtypes in PRISm remained unclear. In this study, we showed that PRISm can be split into two subgroups according to the presence or absence of a restrictive spirometric pattern by assessing their risk factors, baseline lung function, and longitudinal trajectory; one is the restrictive subtype and the other is the obstructive subtype. The pathophysiology of the two types of PRISm remains unclear because detailed examinations, such as computed tomography, post-bronchodilator spirometry, and measurement of total lung capacity, were not performed in this study. However, since non-restrictive PRISm was independently associated with asthma and smoking history, its pathophysiology may encompass intrapulmonary factors, such as airway inflammation, bronchial hyperresponsiveness, and emphysema. In contrast, restrictive PRISm was associated with a high BMI, and thus its pathophysiology may be explained by extrapulmonary factors, such as obesity. The varied pathophysiology may underlie the differences in risk of transition to airflow limitation between the two types of PRISm. Further investigation is warranted to clarify the pathophysiology, incidence of cardiovascular events, and mortality among the two types of PRISm.

In the present study, approximately half of the participants with restrictive and non-restrictive PRISm transitioned to the no PRISm/airflow obstruction category after five years. Previous studies have shown that PRISm is a fluctuating state^6,29,30^. The Copenhagen City Heart Study showed that more than half of the individuals with PRISm transitioned to normal spirometry after 15 years^6^. In the cohort study of Japanese patients, one-third of the individuals with PRISm transitioned to normal spirometry after 3 years^29^. Similar to the previous findings, our results also suggest that PRISm may be a fluctuating state, even if it is classified into two subgroups according to the presence or absence of a restrictive spirometric pattern.

The strengths of our study are the large sample size and the use of multivariate regression to adjust for confounders. However, several limitations should be considered. First, we did not perform post-bronchodilator spirometry because the study population underwent only a general check-up. For the same reason, we did not measure total lung capacity. In the absence of a direct measurement of total lung capacity, it is difficult to evaluate whether the restrictive spirometric pattern actually identifies a true pulmonary restriction^31–33^. A decreased FVC may show a true restrictive spirometric pattern or may reflect airflow obstruction due to air trapping. About 80% of the study participants were male, therefore, gender bias was a limitation of this study. Additionally, we excluded participants who could not be followed-up with 5 years post-initial visit in the longitudinal analysis. This may have led to selection bias. Finally, despite the large sample size of the overall population, there were few individuals who transitioned to airflow obstruction from PRISm.

In conclusion, PRISm can be divided into two subgroups according to the presence or absence of a restrictive spirometric abnormality. One is the restrictive subtype and the other is PRISm without restrictive abnormality; the latter can be classified as normal in the conventional spirometric criteria but is associated with an increased risk of developing airflow obstruction. These findings suggest the need for the stratified management of individuals with PRISm.

## Supplementary Information

Supplementary Information.

## Data Availability

The datasets used and/or analyzed during the current study are available from the corresponding author upon reasonable request.
