# Supplementary material for: Preserved ratio impaired spirometry with or without restrictive spirometric abnormality
Source: Sci Rep. 2023 Feb 20;13:2988. doi: 10.1038/s41598-023-29922-0 (PMC9941093; doi:10.1038/s41598-023-29922-0)

**Supplementary Information**

**Title:** Preserved ratio impaired spirometry with or without restrictive spirometric abnormality

**Authors’ full names:**

Shinichiro Miura^1^, Hiroshi Iwamoto^1^, Keitaro Omori^2^, Kakuhiro Yamaguchi^1^, Shinjiro Sakamoto^1^, Yasushi Horimasu^1^, Takeshi Masuda^1^, Shintaro Miyamoto^1^, Taku Nakashima^1^, Kazunori Fujitaka^1^, Hironobu Hamada^1^, Akihito Yokoyama^3^, Noboru Hattori^1^

**Authors’ affiliation(s):**

^1^Department of Molecular and Internal Medicine, Institute of Biomedical & Health Sciences, Hiroshima University, Hiroshima, Japan

^2^Department of Infectious Diseases, Hiroshima University, Hiroshima, Japan

^3^Department of Respiratory Medicine and Allergology, Kochi University, Kochi, Japan

**Supplementary Table S1. Baseline characteristics of the participants in the longitudinal analysis**

|  |  | No PRISm/airflow obstruction  (n = 1958) | Non-restrictive PRISm (n = 50) | Restrictive PRISm (n = 80) | Airflow obstruction  (n = 53) |  |
| --- | --- | --- | --- | --- | --- | --- |
|  | Characteristics |  |  |  |  |  |
|  | Male, n (%) | 1636 (83.6) | 42 (84.0) | 65 (81.3) | 49 (92.5) |  |
|  | Age (years) | 47.9 ± 6.2 | 48.0 ± 6.1 | 49.5 ± 5.6 | 50.2 ± 6.1 |  |
|  | BMI (kg/m²) | 23.4 ± 3.1 | 23.6 ± 3.2 | 24.1 ± 3.7 | 23.3 ± 2.7 |  |
|  | BMI ⩾ 25, n (%) | 521 (26.6) | 16 (32.0) | 28 (35.0) | 11 (20.8) |  |
|  | BMI ⩾ 30, n (%) | 51 (2.6) | 2 (4.0) | 6 (7.5)* | 1 (1.9) |  |
|  | Smoking status, n (%) |  |  |  |  |  |
|  | Never smoker | 842 (43.0) | 13 (26.0) | 34 (42.5) | 11 (20.8) |  |
|  | Ever smoker with <10 pack-years | 235 (12.0) | 6 (12.0) | 6 (7.5) | 5 (9.4) |  |
|  | Ever smoker with ⩾10 pack-years | 881 (45.0) | 31 (62.0) | 40 (50.0) | 37 (69.8)* |  |
|  | Exposure to dust, n (%) | 178 (9.1) | 2 (4.0) | 4 (5.0) | 2 (3.8) |  |
|  | Cardiac disease, n (%) | 36 (1.8) | 1 (2.0) | 2 (2.5) | 3 (5.7) |  |
|  | Respiratory symptoms, n (%) |  |  |  |  |  |
|  | Cough | 207 (10.6) | 6 (12.0) | 12 (15.0) | 10 (18.9) |  |
|  | Phlegm | 253 (12.9) | 12 (24.0) | 9 (11.3) | 16 (30.2)* |  |
|  | Breathlessness | 536 (27.4) | 24 (48.0)* | 29 (36.3) | 20 (37.7) |  |
|  | History of asthma, n (%) | 154 (7.9) | 8 (16.0) | 7 (8.8) | 15 (28.3)* |  |
|  | Lung function measurements |  |  |  |  |  |
|  | FEV_1_ (L) | 3.23 ± 0.56 | 2.47 ± 0.33*^¶^ | 2.32 ± 0.36* | 2.48 ± 0.60* |  |
|  | %FEV_1_ | 99.4 ± 10.7 | 76.4 ± 2.6*^¶^ | 73.2 ± 4.8* | 73.6 ± 14.2* |  |
|  | FVC (L) | 3.96 ± 0.70 | 3.34 ± 0.44*^¶^ | 2.91 ± 0.46* | 3.78 ± 0.85 |  |
|  | %FVC | 98.4 ± 10.6 | 83.4 ± 2.7*^¶^ | 74.1 ± 4.9* | 89.9 ± 15.3* |  |
|  | FEV_1_/FVC (%) | 81.8 ± 5.1 | 74.1 ± 2.8*^¶^ | 79.8 ± 4.6* | 65.4 ± 4.4* |  |

Variables are presented as mean ± SD or No. (%).

*P < 0.0125 for comparison with No PRISm/airflow obstruction, ^¶^P < 0.0125 for comparison with Restrictive PRISm. Mann–Whitney U-test for continuous and chi-square for categorical variables.

BMI, body mass index; FEV_1_, forced expiratory volume in 1 s; %FEV_1_, percent predicted FEV_1_; FVC, forced vital capacity; %FVC, percent predicted FVC; PRISm, preserved ratio impaired spirometry.

**Supplementary Figure S1. Transitions of lung function categories among participants with restrictive and non-restrictive PRISm between the first visit and the visit after five years**

The distribution of spirometric results of participants with restrictive and non-restrictive PRISm is shown using the conventional criteria of obstructive and restrictive ventilatory abnormalities.

FEV_1_, forced expiratory volume in 1 second; FVC, forced vital capacity; PRISm, preserved ratio impaired spirometry.


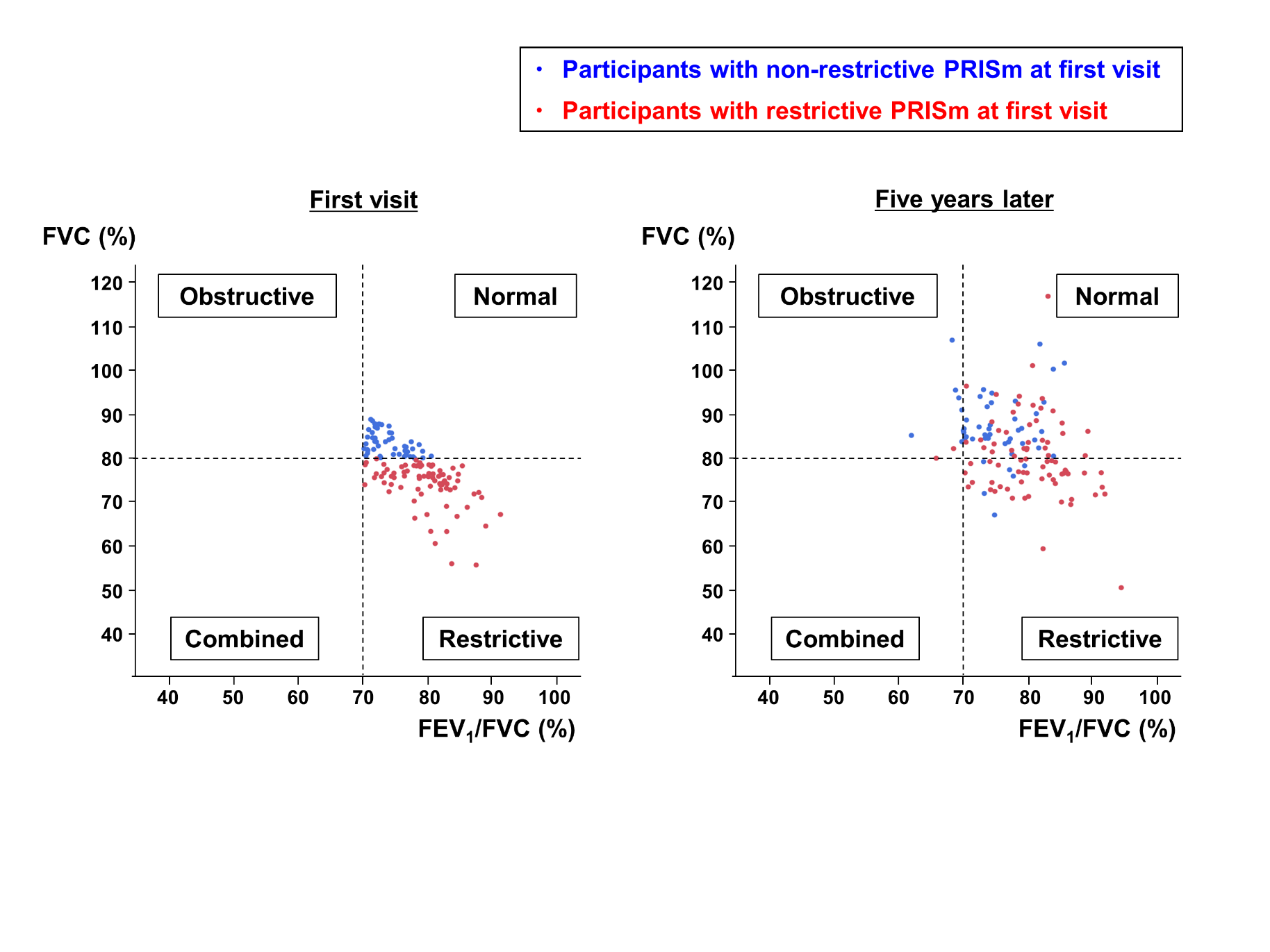

Supplement: Supplementary file 1 — Supplementary Information. [file 41598_2023_29922_MOESM1_ESM.docx]
